# Dietary Supplement Use and Doping Attitudes: A Cross-Sectional Survey

**DOI:** 10.3390/sports14040131

**Published:** 2026-03-26

**Authors:** Amund Riiser, Liam Paul Satchell, Dominic Sagoe, Vidar Andersen, Tom Erik Jorung Solstad, Fredrik Lauritzen, Matthew Shaw

**Affiliations:** 1 Department of Sport, Food and Natural Sciences, Western Norway University of Applied Sciences, 6856 Sogndal, Norway; vidar.andersen@hvl.no (V.A.); tom.erik.jorung.solstad@hvl.no (T.E.J.S.); matthew.peter.shaw@hvl.no (M.S.); 2 Department of Psychology, University of Winchester, Winchester SO22 4NR, UK; liam.satchell@port.ac.uk; 3 Department of Psychosocial Science, University of Bergen, 5020 Bergen, Norway; dominic.sagoe@uib.no; 4 Human Enhancement and Body Image Lab (HEBI Lab), Addiction Research Group, University of Bergen, 5020 Bergen, Norway; 5 Science and Medicine, Anti-Doping Norway, 0855 Oslo, Norway; fredrik.lauritzen@antidoping.no

**Keywords:** drug, incremental model of doping behavior, performance, nutrition, performance enhancement, athletes

## Abstract

Background: The incremental model of doping behavior (IMDB) posits that doping develops over time through the habit of using performance enhancers such as dietary supplements. We investigated the association between dietary supplement use and beliefs and doping attitudes among Norwegian sportspersons. Methods: A total of 1441 subjects (females: 44%; age 31.3 ± 11.6 years) responded to an online questionnaire including measures of dietary supplement use and beliefs, performance enhancement attitude (PEAS), and a doping likelihood vignette. Data were analyzed using descriptive statistics, correlations, and multiple regression analysis. Results: 58% used dietary supplements. Dietary supplement beliefs were positively correlated with doping attitudes (*r* = 0.27 (PEAS) and *r* = 0.16 (vignette), *p* < 0.001). Among non-competitive respondents, younger respondents were more likely to endorse supplement use (*r* = −0.08, *p* = 0.073 vs. *r* = −0.30, *p* < 0.001) and doping use (*r* = −0.17, *p* < 0.001 and *r* = −0.21, *p* < 0.001). Males endorsed supplement use (Welch’s *t* tests > 5.19, *p* < 0.001) and doping (Welch’s *t* tests > 4.08, *p* < 0.001) more than females. Norwegian sportspersons are generally ambivalent about dietary supplements but opposed to doping practices in sport. Results of multiple regression analysis indicated that younger, male, non-competitive, and supplement-endorsing participants were more likely to endorse doping likelihood. However, these differences were small, and participants were generally against doping. Conclusions: The associations between dietary supplement use and beliefs and doping attitudes are weak but compatible with the IMDB. The differences between groups are small; however, focusing on beliefs about dietary supplements in young, male, non-competitive persons may improve the effectiveness of anti-doping interventions.

## 1. Introduction

The World Anti-Doping Agency (WADA) was established in 1999 to promote clean sport worldwide. WADA is responsible for developing the World Anti-Doping Code (WADC), which aims to prevent the use of performance-enhancing drugs among athletes by harmonizing anti-doping policies, rules and regulations within the world of sport [1]. The Code, together with eight international Standards constitute the World Anti-Doping Program. Together they include, among other things, anti-doping rules, a list of prohibited substances and methods, testing procedures, sanctions for violations, and other measures designed to protect the health and safety of athletes. Many international sport federations and national governments have adopted and implemented the WADC into their own anti-doping policies and regulations.

Norway is considered a pioneer in anti-doping work, with WADA recognizing the country as one of the most effective anti-doping organizations in the world [2,3]. Since it was established in 2003, the Norwegian Anti-Doping Agency (Anti-doping Norway—ADNO) has extended its mandate and is currently committed to working both for clean sport and a doping-free society [4]. Norwegian governments have long considered doping an issue that extends beyond the sports arena into a societal and public health issue [2,5]. In 2013, Norway enacted legislation against the use of doping substances such as anabolic-androgenic steroids. Anti-doping Norway is one of a few anti-doping organizations in the world to perform doping testing in commercial fitness centers [6], provide anti-doping education in public and educational institutions [7] and, recently, in prisons, and deploy novel preventive anti-doping interventions in these settings [8]. The unique focus on anti-doping may have affected Norwegian sports persons attitudes and beliefs about performance-enhancing substances.

Hurst et al. [9,10,11,12] have extensively investigated attitudes toward doping by examining the relationship between dietary supplement use and doping attitudes. Two of these studies [10,11] have demonstrated that supplement use is related to more positive attitudes towards and increased likelihood of doping, supporting the hypothesis that supplements serve as a ‘gateway’ to doping [13,14]. The literature suggests that supplement users are an ‘at-risk’ group for potential doping offenses, and anti-doping organizations should include education around dietary supplements [15]. Furthermore, it is well known that dietary supplements may contain both declared and undeclared prohibited substances [16,17]. In support of this, in 26% of all 192 analytic doping rule violations under ADNO’s national doping control program in Norwegian sport from 2003 to 2020, the athletes claimed that a dietary supplement was the source of the prohibited substance detected in the athletes’ biological sample [18], leading to the conclusion that dietary supplements are a major cause of anti-doping rule violations.

The incremental model of doping behavior (IMDB) [19] suggests that doping is a learned behavior developed over time from the habit of using performance-enhancing methods such as dietary supplements [12]. IMDB theories doping instrumentally as a means of improving an individual’s athletic performance, rather than cheating and gaining unfair advantages, and is driven by pressure to perform, rationalization and ethical drift. In critique of this, Hurst et al. [12] have recently extended their existing research to include analyses of moral behavior, concluding that personal morality moderates the indirect relationship between sport supplement use and doping use via sport supplement beliefs, even when individuals believe supplements are necessary for performance. Thus, doping is a moral choice rather than a functional one. Christiansen et al. have investigated doping practices in recreational athletes [20] and suggest that the mechanisms leading to doping are different in recreational athletes compared to elite athletes.

Tangen and Møller [4] argue that the policies and practices of ADNO are reflective of a Nordic social-democratic welfare ideology, whereby doping challenges the Norwegian self-image values of being good, responsible and equal. Norway is a self-defined moral leader [3] in combating doping in sport, and this reflects a morality that permeates all aspects of Norwegian culture. This provides a rationale for exploring this population and their attitudes towards doping. Given the few exploratory studies on doping and doping substance use in Norway [3,21,22], there is currently a paucity of research on Norwegians’ attitudes towards doping.

Norway, therefore, presents an interesting setting for examining attitudes towards doping. The combination of extensive national anti-doping work and a historically egalitarian culture of fairness and “cleanness” suggests that IMDB may be less relevant and Norwegians may respond differently to the findings from Hurst et al. [10,11]. However, dietary supplement use is increasing [23], and it is unclear whether the violations analyzed by Lauritzen [18] are related to unintentional or intentional doping. Thus, we set out to test the hypothesis that dietary supplement use and beliefs are positively associated with favorable doping attitudes among Norwegian sportspersons. We hypothesize that non-competitors, males, and younger sportspersons (compared to competitors, females, and older sportspersons) were more likely to endorse doping use.

## 2. Materials and Methods

### 2.1. Design and Procedure

**Figure 1:**
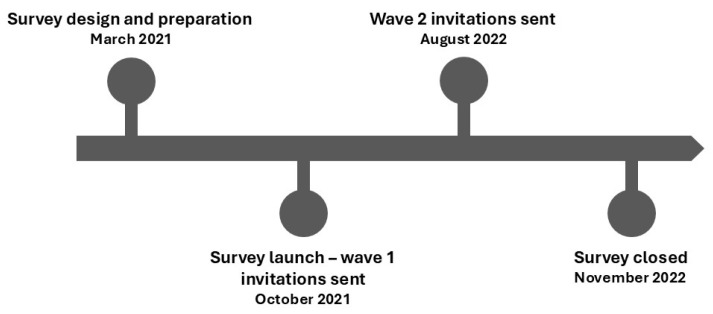
Timeline for the survey.

This cross-sectional study is based on an online survey including a vignette. Scales originally published in English (Sports Supplements Belief Scale and Performance Enhancement Attitude Scale) were translated into Norwegian using a back-translation process [24]. The bilingual members of the author team provided an initial translation from English to Norwegian. Upon agreement on the Norwegian items, an independent professional translator translated the items back into English. A second independent professional translator then reviewed the back-translated items and resolved discrepancies. The final items were used in the survey. The link to the survey was shared via open social media sites related to sport and exercise and via a QR code on posters in various sports centers. The link was also distributed to sport students through official learning platforms at different Norwegian high schools and universities within the authors’ professional networks. The survey was open from October 2021 to November 2022 (Figure 1). Inclusion criteria were that respondents had to be over the age of 18 and report regular participation in sport and exercise activities. Upon opening the electronic survey, participants were directed to the participant information section. This included information about the study, their right to withdraw from the study, the time to complete the survey (5–7 min), and that valid consent was assumed if they partly or fully completed the questionnaire (Supplementary Materials File S1). The study was conducted in line with the Declaration of Helsinki and was approved by the ethical committee of the faculty.

### 2.2. Participants

In total, there were 1624 engagements with the survey link. Respondents with entirely blank responses (*n* = 146) were excluded from further analysis. Others (*n* = 37) were excluded due to reporting an age under 18 and, therefore, not meeting the inclusion criteria. Thus, 1441 respondents who answered some or all questions were included. The sample was reasonably split between male and female participants (the small number of other gender responses were not included in further analysis; see Table 1). A total of 1356 respondents, ranging in age from 18 to 76 (*M* = 31.3, *SD* = 11.6), reported their highest level of competition. A total of 1234 respondents answered the Sports Supplements Belief Scale and the Performance Enhancement Attitude Scale. Table 1 and Table 2 present additional sample characteristics.

### 2.3. Measures

The online survey was conducted using a questionnaire assessing the following variables.

#### 2.3.1. Demographics

Respondents indicated their sex (man, woman, other, prefer not to say), age (years), and highest completed education (secondary school, high school, college, bachelor, master, PhD).

#### 2.3.2. Competition Level

Competition level was assessed using the question “What is the highest level you have competed at? (During the last three years). Response options were “do not compete, recreational, local, regional, national, international”.

#### 2.3.3. Dietary Supplement Use

Participants responded to the question “Do you take dietary supplements?” (yes/no). Those responding yes subsequently answered the question “Which kind(s) of dietary supplements do you take?” selecting from the following list: protein/amino acids, caffeine, creatine, gainers, carbohydrates (sports drink), pre-workout, fat burners, vitamins and minerals, superfoods, testosterone boosters or other.

#### 2.3.4. Dietary Supplements Beliefs

Respondents who reported using dietary supplements completed the Sports Supplements Belief Scale [9]. The SSBS consists of six items. As our sample contained non-competitive respondents, we used a tailored version of the SSBS for their general outcomes. The modifications were to ensure that items pertinent to competing and winning were reworded so that they referenced achieving one’s goals. Dietary supplement-using competitive respondents completed the standard SSBS (e.g., “My chances of winning improve by using supplements”), whereas dietary supplement-using non-competing respondents completed the modified SBSS (e.g., “My chances of achieving my goals are improved by using dietary supplements”). For each version, level of agreement is indicated on a six-point Likert scale (1 = completely disagree, 6 = completely agree). The SSBS was not validated after the modification. However, we consider it to increase the external validity. Stil comparison with studies including non-competitive athletes using the original SSBS should be performed with caution. We did not present the SSBS to athletes not taking dietary supplements, as the items in the SSBS inquire about the experience of using supplements, and that it is not relevant if you have not taken dietary supplements. In our data, the internal consistency for the competing (Cronbach’s α = 0.87, McDonald’s ω = 0.88) and non-competing (α = 0.88, ω = 0.89) respondents to the SSBS was good. A one-factor confirmatory factor analysis supported that the scale was all measuring the same construct (TLI= 0.94, CFI= 0.97). In line with previous research, the mean response to the items was retained for analysis.

#### 2.3.5. Performance Enhancement Attitude

To assess doping attitudes, respondents completed the 8-item version of the Performance Enhancement Attitude Scale (PEAS) [25]. The response scale was again on a six-point Likert scale ranging from completely disagree (1) to completely agree (6). The internal consistency for this scale was adequate (α = 0.75, ω = 0.80). There was notable skewness in responding across the items (average skew for all 8-items was 2.23), with respondents frequently strongly rejecting the items. This was most strongly seen in the items ‘[doping] is not cheating since everybody does it’ (skew = 3.32, 81% of respondents selecting completely disagree) and ‘Athletes should not feel guilty about breaking the rules and taking performance-enhancing drugs.’ (skew = 3.54, 80% completely disagree).

In line with previous research, we retained average response to the PEAS for analysis. However, to account for these skewed responses, we additionally retained the total number of respondents’ non-completely disagree responses across the PEAS scale for analysis. Any response scoring greater than ‘1’ was considered a non-complete rejection. The total number of items out of the eight which did not receive complete rejections was summed as a ‘PEAS endorsement’. We did this as the norm response for participants was to reject the PEAS items, and therefore, the interesting difference in this data is between those who outright rejected the items versus those who showed some endorsement. The sample was largely treating the scale in a dichotomous manner, with distinct response patterns breaking into complete rejection versus minor endorsement. We include both forms of analysis using average PEAS and our computed ‘PEAS endorsement’ for completeness, but with an applied focus, it is important to consider the informativeness of non-complete rejections—people who might consider doping behaviors acceptable in some situations. The resulting non-complete rejection PEAS variable was more suitable for statistical analysis, having a normal distribution (skew = 0.73, min = 0, max = 8).

#### 2.3.6. Doping Likelihood (Vignette Measure)

To assess the likelihood of doping, participants responded to a vignette. Respondents participating in competitions were presented with a vignette used in previous research [10,26]. For competitive respondents, the vignette focused on the benefits of using a banned substance to improve fitness and performance for a future competition. The following vignette was used:

*It’s the week before the most important competitive game/event of your season. Lately, your performance has been below your best. You don’t feel you have the necessary fitness for this competition, and you’re concerned about how you’ll perform. You mention this to a teammate, who tells you that he/she uses a new substance that has enhanced his/her fitness and performance. The substance is banned for use in sport, but there’s no chance that you will be caught.*

Respondents not participating in competitions were presented an adapted vignette focused on the benefits of using a banned substance to improve fitness and body image. The adapted scenario is presented below:

*Recently, you have not been able to train/respond to your training as you had planned/hoped. You feel like your body doesn’t look or perform the way it should. You mention this to a training buddy who tells you that he/she is using a new drug that has made it easier to achieve results. The drug is forbidden to use, but there is no possibility of you being caught.*

After reading the vignette, respondents rated how likely they were to use the banned substance on a Likert-type scale ranging from 1 (not at all likely) to 7 (very likely).

A version of all questions translated from Norwegian to English by Google Translate can be found as Supplementary Materials File S1.

### 2.4. Statistical Analysis

Descriptive statistics comprising frequencies and means were used to ascertain sample characteristics. Group differences were identified primarily through inference from *p* values (computed through Welch’s *t* tests and Pearson’s correlations), effect sizes, and overlap coefficients. For tests of relationships, we used correlations and drew inferences from size of effect and *p* values. The overlap coefficient describes the proportion of respondents which present with the same range in the data set and can be read as how distinguishable two response distributions are [27,28]. The higher percentage of overlap, the less meaningful difference there is between groups. It was also of interest to model the predictors of the vignette endorsement from a multivariate perspective. We simultaneously entered the demographic (age, sex, and competitive level) and supplement use (SSBS) measures as predictors of the vignette response. The R packages psych [29], jmv [30], and overlapping [31] were primarily used for the analyses in R version 4.4.0. We used one-sample *t* tests to compare means from our data and other published data. Due to running several tests on one sample, we draw inference from a conservative *p* < 0.001 throughout.

## 3. Results

### 3.1. Prevalence of Supplement Use

In our sample, 58% of participants reported using supplements of some form (females: 55%, males: 60%). Among competitive and non-competitive participants, 59% and 57% reported supplement use, respectively. Most respondents reported using vitamins and minerals (38%), followed by caffeine (35%), protein/amino acids (31%), creatine (27%), “pre-workout” (14%), carbohydrates (in sports drink form, 8%), “superfoods” (2%), “fat burners” (2%), “gainers” (2%), and testosterone boosters (1%). Of those reporting supplement use, 8% used one supplement, 32% used two supplements, and 21%, 20%, and 15% used three, four, and five supplements, respectively.

### 3.2. Supplement Beliefs and Performance Enhancement Attitude

Supplement users tended to provide mid-scale responses on the SSBS (mean 3.53, *SD* 1.06), indicating partial agreement or partial disagreement with the items. There were no differences in supplement beliefs between competitors and non-competitors (*p* = 0.18). In contrast, the whole sample’s mean responses on the PEAS were 1.66 (*SD* 0.65), often representing complete disagreement or disagreement. There were statistically significant differences (*p* < 0.001) in PEAS responses between competitors (mean 1.60, *SD* 0.61) and non-competitors (mean 1.76, *SD* 0.69), and between supplement users (mean 1.73, *SD* 0.70) and non-users (mean 1.57, *SD* 0.54). On average, the sample made three endorsements (non-complete rejections) across the eight PEAS items; see Table 2.

### 3.3. Doping Likelihood

**Figure 2:**
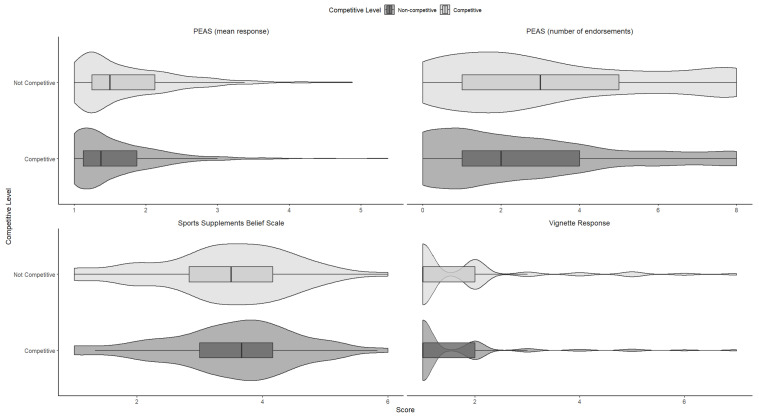
Violin plots to demonstrate the difference and overlap between the self-reported competitive and non-competitive respondents on the SSBS, PEAS (mean and endorsement responses) and the vignette responses. The figures should show the skew in the vignette and PEAS responses.

Also present in Table 1 is how the sample engaged with the hypothetical doping vignettes. The results were in line with the questionnaire measures, with non-competitors (mean 1.82, *SD* 1.34) compared to competitors, (mean 1.59, *SD* 1.18) and supplement users (mean 1.83, *SD* 1.24) (as opposed to non-users, mean 1.46, *SD* 0.92) were more likely (<0.01) to endorse the hypothetical scenario. Nearly all cases respondents selected “very unlikely” (23%) or “definitely not” (64%, for a combined 87%). Where there were differences between the groups, these were with small effect sizes, SDs as large as the means, and with 87% of responses across groups overlapping (see Table 1 and Figure 2).

### 3.4. Correlations Between Supplement and Doping Measures

Table 3 presents the correlations between the outcome variables in this study. There were positive correlations between SSBS, PEAS and vignette measures.

### 3.5. Correlations with Age and Sex

Table 4 presents the correlations between age and responses to the scales by participant group. There was no significant correlation betweenage and supplementbeliefs (*r* = −0.08, *p* = 0.07). Age correlated negatively with doping endoresement (*r* = −0.21 − −17, *p* < 0.01. However, for non-competitive respondents, there were negative correlations between age and endorse supplement beliefs (*r* = −0.31, *p* < 0.001) and doping behaviours (*r* = −0.22 − −13, *p* < 0.001).

The sex differences between respondents’ responses can be found in Table 5. There were notable differences throughout, with male respondents being more likely to endorse supplements and doping behaviors. This was particularly distinct in the non-competitive supplement user groups, where there were significant differences between male and female participants.

### 3.6. Multivariate Prediction of Vignette Response

Based on the above findings, a multivariate model assessing the ability of demographics and supplement belief to explain responses to the vignette was conducted. The visual inspection of Q-Q plots and residual histograms showed acceptable data for modeling (with skews at extremes, as is expected in large sample data). This model explained 8% of the variance (*R*^2^_Adj_= 0.08, *F* [10,696] = 6.99, *p* < 0.001) with all predictor VIF ≤ 1.11. The significant predictors were the same as above, with younger (β_unstandardised_ = −0.02, β_standardised_ *=* −0.12, 95% CI [−0.12, −0.11], *p* = 0.002), male (β_unstandardised_ = 0.32, β_standardised_ *=* 0.12, 95% CI [−0.10, 0.33], *p* = 0.003), non-competitive (β_unstandardised_= 0.45 β_standardised_ *=* 0.15, 95% CI [−0.05, 0.36], *p* < 0.001) and supplement endorsing (β_unstandardised_= 0.16, β_standardised_ *=* 0.12, 95% CI [0.02, 0.22], *p* = 0.001) participants being more likely to endorse doping in the vignette. This reflects the above findings, but there was an overall limited amount of variance explained.

### 3.7. Comparisons to Extant Data

The SSBS score in our study was 3.49 (*SD* = 1.07) and higher *(t* [743] = 12.21, *p* < 0.001, *d* = 0.45) compared to 3.01 (*SD* = 1.12) in the study by Hurst et al. [10]. The PEAS score was significantly lower in our study (*M* = 1.66, *SD* = 0.65) than Hurst et al.’s findings (*M* = 2.09, *SD* = 0.82, *t* [1440] = 29.97, *p* < 0.001, *d* = 0.80). The same trend with less endorsement of doping in our sample was evident for the scenarios where the doping scenario scores were lower in our sample (*M* = 1.68, *SD* = 1.24, *t* [1219] = 16.70, *p* < 0.001, *d* = 0.48) compared to 2.27 (*SD* = 1.53) for Hurst et al. [10]. This is also the case when looking at the competitors in our sample alone, like Hurst et al.’s sample (*M*= 1.82, *SD* = 1.34, *t* [765] = 15.43, *p* < 0.001, *d* = 0.58). Interpretation of this difference should be treated with caution due to linguistic differences between the scales.

## 4. Discussion

The present study examined the relationship between dietary supplement use, beliefs, and doping attitudes among Norwegian sportspersons.

### 4.1. Supplement Use and Beliefs, and Doping Attitudes and Likelihood

Supplement beliefs did not meaningfully differ between competitive and non-competitive respondents, with no significant difference and 95% overlap in their responses. The SSBS score in our study was significantly higher than the SSBS score in the study by Hurst et al. [10]. However, we opted not to present the SSBS to athletes not taking dietary supplements, as the items in the SSBS inquire about the experience of using supplements, and that it is not relevant if they have not taken dietary supplements. An estimated 49% of the athletes in the study by Hurst et al. [32] did not take supplements, and athletes not taking supplements are shown to score lower on the SSBS [9]. It is therefore difficult to compare the supplement beliefs in the two studies.

For the PEAS measures (by both metrics), there was some evidence that competitors were less likely to endorse doping than non-competitors. However, it should be noted that the effect size of this difference was small, and there was 90–91% overlap in the responses provided by these groups. Similarly, there was a significant difference between those who use supplements and those who do not on their PEAS scores (both metrics), with more doping endorsement by supplement users. However, again, the effect size of the difference was small, and there was 87–89% overlap in the responses provided by participants, indicating that few individuals were driving the difference between groups.

To our knowledge, no previous study has investigated the differences in doping endorsement between competing and non-competing athletes, and the reason for this difference remains unknown. However, Christiansen has investigated doping and gym culture, suggesting that the psychological mechanisms, social pressure and cultural influence lead to doping being far more pervasive in gym goers than elite athletes [20,33]. Furthermore, a recent study on attitudes toward doping use among Norwegian adolescents reports a higher acceptance and intention to use doping substances for increasing muscle mass and losing weight, compared to a lower acceptance for doping use for increasing performance in sports [34]. We also speculate that the reduced risk of being tested when not competing and the fact that you do not cheat anyone but yourself may cause more doping endorsement in non-competing athletes. The more liberal attitudes to doping in non-competing athletes also fit with the hypothesis that fitness doping is increasingly being transferred into mainstream fitness and gym culture [35], probably representative of some of our included athletes as we recruited participants through fitness centers in addition to other sources. This would suggest other possible explanations for doping than the IMDB.

Our study suggests that Norwegian athletes are moderately positive about dietary supplements, but against doping practices in sport. There was some evidence that athletes who exercised recreationally and did not train to compete were more likely to endorse doping than competing athletes, as well as evidence that those engaged in supplement use were more likely to endorse doping. However, it should be noted that these differences were negligible, and participants were all against doping in general. These results were consistent between the use of validated questionnaires and hypothetical vignettes.

Thus, our findings suggest that Norwegian athletes have a strong rejection of doping compared to other athletes. Norway’s early and long-term commitment to anti-doping work [2,3] and the ADNO strategy to work against doping on a broad scale and Norwegian egalitarian culture [4] may have contributed to these attitudes.

In the present study, supplement use was positively associated with attitudes to doping and the likelihood of doping. These results are in line with previous research from the USA [14] and the UK [13]. However, Hurst et al. [10] did not find a direct association between dietary supplement use and attitudes to doping assessed with the SSBS or doping likelihood assessed with scenarios in competitive athletes in the UK. However, they reported an association mediated by sport supplement beliefs.

Our study found that there was a weak association between athletes’ dietary supplement beliefs and doping attitudes. Athletes who were more likely to endorse supplement use were more likely to score higher on the PEAS measures and more likely to support the doping scenario. These findings are supported by Hurst [10,11], who demonstrated that dietary supplement beliefs mediate the relationship between dietary supplement use and doping attitudes. The associations between dietary supplement beliefs and doping attitudes were weak in our study, and we suspect that the extensive national anti-doping work and a historically egalitarian culture of fairness and “cleanness” may partly explain why Norwegians respond differently compared to Hurst et al. [10,11] participants. However, Hurst et al. [10] also found a weak association between dietary supplement beliefs and the likelihood of doping.

### 4.2. Associations with Age and Gender

Given the variability in age and gender in our sample, we were able to analyze whether these demographic variables had an impact on the variables in this study. With the exception of supplements, belief in competitive supplement users, supplement belief, attitudes to doping, and the likelihood of doping were stronger in younger athletes. There were notable differences between the genders, with male respondents being more likely to endorse supplements and doping behaviors. This is in line with records from the doping control program in Norwegian sport from the period 2003–2019, where male athletes accounted for 97% of the anti-doping rule violations in the period [36], and with a national study among Norwegian adolescents where males more frequently report doping use, have more liberal attitudes towards doping substances and a higher intent to use [34]. The difference was particularly distinct in the non-competitive supplement user groups, where there were significant differences in the distribution of responses from male and female participants. Research shows clear gender differences in both doping prevalence and attitudes, possibly explained by women displaying greater moral opposition to doping and more concern about health risks than men [37]. Thus, it seems important that anti-doping work focuses on male recreational exercisers not taking part in athletic competition.

### 4.3. Practical Implications

Our research has practical implications for anti-doping practice. Our study confirms that dietary supplement use and positive dietary supplement beliefs are associated with attitudes to doping and likelihood to dope, also in a population of athletes strongly rejecting doping. Thus, anti-doping programs should focus on the risk, the need, and the effect of dietary supplements [17,38], and as the sales of dietary supplements are increasing globally [23,39], this may be increasingly important in the future. Even though some dietary supplements may increase sport performance [39,40,41], athletes using dietary supplements are more likely to respond to a placebo than the actual supplement [11]. Therefore, knowledge about the nutritional and placebo effects of dietary supplements may help athletes make more informed choices about dietary supplement use and modify athletes’ beliefs about their effectiveness. Dietary supplement use is also related to many positive doping tests [18]; thus, the risk of positive doping tests should be weighed against the unproven or minor effects of dietary supplements. Our findings suggest that anti-doping campaigns aiming to reduce doping through altering dietary supplement beliefs should focus on younger athletes, male athletes and non-competitive exercisers. These were more likely to support supplement and doping use.

### 4.4. Strengths and Limitations

The study is strengthened by an adequate sample of 1441 participants and even distribution between supplement users and non-supplement users, competing athletes and not competing athletes, males, and females, which strengthens the external validity of the results. We used validated questionnaires and translated them into Norwegian according to a recommended protocol, which strengthens the internal validity of the results and makes them easier to compare with other studies using the same questionnaire.

However, this is a cross-sectional study; therefore, we are only able to identify associations and not prove causality between supplement use or supplement beliefs and doping attitudes or doping likelihood. The IMBD suggests that doping behavior develops over time; thus, our study design cannot fully assess it. It could be argued that positive attitudes towards doping cause supplement use and beliefs, even if we, based on IMBD, believe it is the other way around, starting with legal perceived performance enhancers and moving on to illegal performance enhancers. The representativity of the sample can also be questioned, as we recruited a convenience sample through multiple strategies over more than a year. Thus, our sample is probably not representative of Norwegian sportspersons and attitudes and beliefs may change over time. This may weaken the external validity of the results. The SSBS is developed for competing athletes, and we tailored the SSBS for their general outcomes. The modifications were to ensure that items pertinent to competing and winning were reworded so that they referenced achieving one’s goals. Thes modification probably made the SSBS more relevant for non-competing athletes, but they may also have influenced the validity of the tool. Even if the survey was anonymous, social desirability bias may influence the responses, particularly as doping is a sensitive topic [42]. The vignettes and the SSBS were modified to be relevant for non-competing athletes. The modified tools are not validated, thus comparison with studies using the original tool should be performed with caution, as we do not know how it affected the responses. If the modification of the tool decreases the accuracy of the measurement, this may have contributed to weaker associations and smaller effects.

## 5. Conclusions

The present study demonstrates that use of and beliefs in dietary supplements are associated with positive attitudes towards doping and the likelihood to dope, even if the athletes are dismissive of doping. There are small differences between the groups, but younger athletes, male athletes and athletes not competing were more positive towards doping. Therefore, anti-doping work may also include information about the effects and risks of using dietary supplements and target young athletes, male athletes and athletes not competing in sports. Our results support the IMBD, indicating that supplement use can also lead to doping in a population generally dismissive of doping. Future research may assess the effectiveness of anti-doping programs focusing on sport supplements, preferably in a population more positive towards doping.

## Figures and Tables

**Table 1:** Sample characteristics.

Category	Subcategory	*n*
Gender	Male	756
	Female	696
	Other	4
	Prefer not to say	5
Highest level of competition	Recreational	174
	Local	99
	Regional	141
	National	268
	International	152
	Total competitive athletes	768
Highest completed education	High school	419
	Bachelor’s degree	406
	Master’s degree	312

**Table 2:** The descriptive statistics for the SSBS, PEAS, PEAS non-rejection totals, and the responses to the vignettes for the whole sample, compared by competitors or not and by supplement use or not (with comparisons using Welch’s *t*, significance, Cohen’s d and the overlap coefficient (*ovl*)).

		*n*	Mean (*SD*)	Difference Welch’s *t* (*p*) [*d*] *ovl*
SSBS (score 1–6)	Competitors	458	3.53 (1.06)	
	Non-Competitors	286	3.42 (1.08)	1.35 (0.18) [0.10] 0.95
	Supplement use	744	3.49 (1.07)	
	No Supplement use	-	-	
	Whole sample	744	3.49 (1.07)	
PEAS (score 1–6) (mean)	Competitors	768	1.60 (0.61)	
	Non-Competitors	466	1.76 (0.69)	4.09 (<0.01) [0.24] 0.90 *
	Supplement use	718	1.73 (0.70)	
	No Supplement use	516	1.57 (0.54)	4.45 (<0.01) [0.25] 0.89 *
	Whole sample	1234	1.66 (0.65)	
PEAS (score 1–6) (Non-rejection)	Competitors	768	2.62 (2.36)	
	Non-Competitors	466	3.29 (2.60)	4.56 (<0.01) [0.27] 0.91 *
	Supplement use	718	3.07 (2.57)	
	No Supplement use	516	2.59 (2.30)	3.38 (<0.01) [0.19] 0.91 *
	Whole sample	1234	2.87 (2.47)	
Response to Vignettes (score 1–7)	Competitors	766	1.59 (1.18)	
	Non-Competitors	454	1.82 (1.34)	3.01 <0.01) [0.18] 0.87 *
	Supplement use	708	1.83 (1.42)	
	No Supplement use	512	1.46 (0.92)	5.65 (<0.01) [0.32] 0.87 *
	Whole sample	1220	1.68 (1.24)	

* Indicates significance at a conservative *p* < 0.01. Sports Supplements Belief Scale: SSBS, Performance Enhancement Attitude Scale: PEAS.

**Table 3:** Correlations between measures used in this study are Pearson’s *r* (*p*) with 95% CI [Lower, Upper].

	PEAS (Mean)	PEAS (Endorsement)	Vignette
SSBS	0.27 (<0.001) * [0.21, 0.34]	0.24 (<0.001) * [0.17, 0.31]	0.16 (<0.001) * [0.09, 0.23]
PEAS (mean)	-	0.85 (<0.001) * [0.83, 0.87]	0.50 (<0.001) * [0.46, 0.54]
PEAS (endorsement)	-	-	0.41 (<0.001) * [0.37, 0.46]

* denotes significance at a conservative *p* < 0.01. Sports Supplements Belief Scale: SSBS, Performance Enhancement Attitude Scale: PEAS.

**Table 4:** Correlations between age and measures used in this study by participant group as Pearson’s *r* (*p*) with 95% CI [Lower, Upper].

	Competitive, Supplement Users	Non-Competitive Supplement Users	Non-Supplement Users
SSBS	−0.08 (0.07) [−0.17, 0.01]	−0.31 (<0.001) * [−0.41, −0.20]	-
PEAS (mean)	−0.16 (<0.001) * [−0.24, −0.07]	−0.20 (<0.001) * [−0.31, −0.09]	−0.21 (<0.001) * [−0.24, −0.09]
PEAS (endorsement)	−0.21 (<0.001) * [−0.29, −0.11]	−0.22 (<0.001) * [−0.33, −0.10]	−0.21 (<0.001) * [−0.29, −0.13]
Vignette	−0.17 (<0.001) * [−0.26, −0.08]	−0.13 (0.04) [−0.24, −0.01]	−0.19 (<0.001) * [−0.27, −0.11]

* denotes significance at a conservative *p* < 0.001. Sports Supplements Belief Scale: SSBS, Performance Enhancement Attitude Scale: PEAS.

**Table 5:** Sex differences in responses to measures used in this study by participant group reported as Welch’s *t* tests (*p* values) [Cohen’s *d*] and the overlap coefficient (ovl).

	Female Respondents	Male Respondents	Difference Welch’s *t* (*p*) [*d*] *ovl*
**Competitive, supplement users**			
SSBS	*n* = 194, *M* = 3.24, *SD* = 1.08	*n* = 261, *M* = 3.75, *SD* = 0.97	5.19 (<0.001) [0.50] 0.83 *
PEAS (mean)	*n* = 183, *M* = 1.42, *SD* = 0.44	*n* = 253, *M* = 1.79, *SD* = 0.72	6.70 (<0.001) [0.63] 0.72 *
PEAS (endorsement)	*n* = 183, *M* = 2.09, *SD* = 2.12	*n* = 253, *M* = 3.18, *SD* = 2.51	4.92 (<0.001) [0.47] 0.82 *
Scenario responses	*n* = 182, *M* = 1.48, *SD* = 1.09	*n* = 253, *M* = 1.79, *SD* = 1.41	2.60 (0.010) [0.25] 0.86
**Non-competitive supplement users**			
SSBS	*n* = 143, *M* = 3.00, *SD* = 1.06	*n* = 142, *M* = 3.85, *SD* = 0.93	7.15 (<0.001) [0.85] 0.69 *
PEAS (mean)	*n* = 133, *M* = 1.59, *SD* = 0.63	*n* = 136, *M* = 2.15, *SD* = 0.78	6.41 (<0.001) [0.78] 0.68 *
PEAS (endorsement)	*n* = 133, *M* = 2.56, *SD* = 2.46	*n* = 136, *M* = 4.59, *SD* = 2.56	6.64 (<0.001) [0.81] 0.65 *
Scenario responses	*n* = 133, *M* = 1.75, *SD* = 1.31	*n* = 135, *M* = 2.44, *SD* = 1.69	3.76 (<0.001) [0.46] 0.71 *
**Non-supplement users**			
SSBS	-	-	-
PEAS (mean)	*n* = 259, *M* = 1.48, *SD* = 0.47	*n* = 263, *M* = 1.67, *SD* = 0.60	4.08 (<.001) [0.36] 0.88 *
PEAS (endorsement)	*n* = 259, *M* = 2.33, *SD* = 2.27	*n* = 263, *M* = 2.89, *SD* = 2.32	2.78 (0.006) [0.24] 0.89
Scenario responses	*n* = 255, *M* = 1.39, *SD* = 0.86	*n* = 256, *M* = 1.52, *SD* = 0.97	1.62 (0.106) [0.14] 0.90

* Denotes significance at a conservative *p* < 0.001. Sports Supplements Belief Scale: SSBS, Performance Enhancement Attitude Scale: PEAS. SSBS has possible scores between 6 and 36, PEAS (mean) has possible scores between 8 and 48, PEAS (endorsement) has possible scores between 0 and 8. Sample sizes vary in this table, though even the smallest sample size comparison and smallest effect (*n* = 133 vs. *n* = 135 for *d* = 0.46 achieved 96% power.

## Data Availability

The datasets used and/or analyzed during the current study are available from the corresponding author on reasonable request.

## Supplementary Materials

The following supporting information can be downloaded at: https://www.mdpi.com/article/10.3390/sports14040131/s1, Supplementary File S1: Translated survey.
