# Supplementary material for: Dietary Supplement Use and Doping Attitudes: A Cross-Sectional Survey
Source: Sports (Basel). 2026 Mar 26;14(4):131. doi: 10.3390/sports14040131 (PMC13119637; doi:10.3390/sports14040131)
Supplement: Supplementary file 1 [file sports-14-00131-s001.zip › sports-4164296-supplementary.pdf]

Translation

Information about the Research Project

Dietary Supplements and Doping

Here you will find information about the aims of this research project and what participation involves for you.

Purpose

In this research project, we aim to learn more about the use of dietary supplements and attitudes toward doping among athletes and others who are engaged in training. Among other things, we will examine the relationship between beliefs about the effectiveness of dietary supplements and attitudes toward doping. We intend to use the results from this project to inform the development of new research projects that will evaluate the effectiveness of Anti-Doping Norway's preventive initiatives.

Who is responsible for the research project?

Western Norway University of Applied Sciences (HVL) is responsible for the project, which is conducted in collaboration with Anti-Doping Norway and Idrettsklynge Vest.

Why are you included in the study?

You are included because you live in Norway and are 16 years of age or older.

What does participation involve?

If you choose to participate, you will be asked to complete an electronic questionnaire. It will take approximately 5–7 minutes. The questionnaire includes questions about you, your use of dietary supplements, and your attitudes toward doping. Your responses will be recorded electronically.

Voluntary and Anonymous Participation

Participation is voluntary, and there will be no negative consequences if you choose not to take part in the survey. No information is collected that can identify who has participated, and your answers cannot be traced back to you as an individual.

If you have questions about the study, would like more information, or wish to exercise your rights, please contact:

Western Norway University of Applied Sciences
Attn: *Amund Riiser*, Amund.Riiser@hvl.no

Our Data Protection Officer:
Trine Anikken Larsen, Trine.Anikken.Larsen@hvl.no

Kind regards,

Amund Riiser

(Associate Professor)

---

(1) I have read the information and wish to participate in the project

What is your age (years)?

_____

Sex

(1) ☐ Male

(2) ☐ Woman

(3) ☐ Other

(4) ☐ Will not answer

What is your highest completed level of education?

(6) ☐ Junior high school

(1) ☐ Secondary school (VGS)

(2) ☐ Annual study

(3) ☐ Bachelor's degree

(4) ☐ Master's degree

(5) ☐ Doctoral degree (PhD)

Which form of training / sport do you spend the most time on?

(the last three years)

____

____

____

____

____

____

How many sessions a week do you spend on this form of training / sport?

(the last three years)

_____

What is the highest level you have competed at?

(the last three years)

(1) ☐ Does not compete

(2) ☐ Exercise competition

(3) ☐ Local

(4) ☐ Regional

(5) ☐ National

(6) ☐ International

Do you take nutritional supplements?

(1) ☐ Yes

(2) ☐ No

What type(s) of dietary supplements do you take?

(6) ☐ Protein and / or amino acids

(1) ☐ Caffeine (coffee, energy drinks, caffeine pills)

(2) ☐ Creatine

(3) ☐ Gainers

(4) ☐ Carbohydrate (sports drink)

(5) ☐ Pre-workout

(7) ☐ Fat burners (CLA, green tea, carnitine, etc.)

(8) ☐ Vitamins and minerals (coconut oil, multivitamin, etc.)

(10) ☐ Superfoods (Goji berries, spirulina, herbs)

(11) ☐ Testo boosters

(9) ☐ Other

How much do you agree / disagree with the following statements about food supplements?

Strongly Disagree Disagree Partly Disagree Partly Agree Agree Strongly Agree

Dietary supplements improve my performance

(1) ☐ (2) ☐ (3) ☐ (4) ☐ (5) ☐ (6) ☐

Dietary supplements are necessary for me to be competitive

(1) ☐ (2) ☐ (3) ☐ (4) ☐ (5) ☐ (6) ☐

Dietary supplements improve my self-esteem

(1) ☐ (2) ☐ (3) ☐ (4) ☐ (5) ☐ (6) ☐

My chances of winning improve when I use supplements

(1) ☐ (2) ☐ (3) ☐ (4) ☐ (5) ☐ (6) ☐

Supplements help me achieve my potential

(1) ☐ (2) ☐ (3) ☐ (4) ☐ (5) ☐ (6) ☐

Supplements improve the quality of my training

(1) ☐ (2) ☐ (3) ☐ (4) ☐ (5) ☐ (6) ☐

How much do you agree / disagree with the following statements about food supplements?

Strongly Disagree Disagree Partly Disagree Partly Agree Agree Strongly Agree

Dietary supplements improve my performance

(1) ☐ (2) ☐ (3) ☐ (4) ☐ (5) ☐ (6) ☐

Dietary supplements are necessary for me to be well enough trained/look good

(1) ☐ (2) ☐ (3) ☐ (4) ☐ (5) ☐ (6) ☐

Dietary supplements improve my self-esteem

(1) ☐ (2) ☐ (3) ☐ (4) ☐ (5) ☐ (6) ☐

The chances of me reaching my goals improve when I use nutritional supplements

(1) ☐ (2) ☐ (3) ☐ (4) ☐ (5) ☐ (6) ☐

Supplements help me achieve my potential

(1) ☐ (2) ☐ (3) ☐ (4) ☐ (5) ☐ (6) ☐

Supplements improve the quality of my training

(1) ☐ (2) ☐ (3) ☐ (4) ☐ (5) ☐ (6) ☐

How much do you agree / disagree with the following claims about doping

Strongly Disagree Disagree Partly Disagree Partly Agree Agree Strongly Agree

There is no difference between performance-enhancing drugs and special equipment that improves performance

(1) ☐ (3) ☐ (5) ☐ (7) ☐ (8) ☐ (9) ☐

The risks of doping are exaggerated

(1) ☐ (3) ☐ (5) ☐ (7) ☐ (8) ☐ (9) ☐

Legalizing performance-enhancing substances will be beneficial for sport

(1) ☐ (3) ☐ (5) ☐ (7) ☐ (8) ☐ (9) ☐

Doping is not cheating because everyone does it

(1) ☐ (3) ☐ (5) ☐ (7) ☐ (8) ☐ (9) ☐

Athletes should not feel guilty about breaking the rules and using performance-enhancing drugs

(1) ☐ (3) ☐ (5) ☐ (7) ☐ (8) ☐ (9) ☐

Only the achievement should matter, not how the athlete achieves it

(1) ☐ (3) ☐ (5) ☐ (7) ☐ (8) ☐ (9) ☐

Doping is necessary to be competitive

(1) ☐ (3) ☐ (5) ☐ (7) ☐ (8) ☐ (9) ☐

Doping is an inevitable part of competitive sport

(1) ☐ (3) ☐ (5) ☐ (7) ☐ (8) ☐ (9) ☐

Imagine the following scenario:

It is the week before the most important match/competition of the season. Lately you have performed worse than your best. You feel that your form is not good enough and you are worried about how you will perform. You mention this to a teammate who tells you that he/she is using a new drug that improves form and performance. The medication is forbidden to be used in connection with sports, but there is no possibility that you will be caught.

How likely is it that you would use doping?

(1) ☐ Absolutely certain (2) ☐ Very likely (3) ☐ Somewhat likely (4) ☐ Not sure (5) ☐ Somewhat unlikely (6) ☐ Very unlikely (7) ☐ Definitely not

Imagine the following scenario:

Recently, you have not trained/responded to your training as you had planned/hoped. You feel that your body does not look or perform as it should. You mention this to a training buddy who tells you that he/she is using a new drug that has made it easier to achieve results. The medication is forbidden to use, but there is no chance of you being caught.

How likely is it that you would use doping?

(1) ☐ Absolutely certain (2) ☐ Very likely (3) ☐ Somewhat likely (4) ☐ Not sure (5) ☐ Somewhat unlikely (6) ☐ Very unlikely (7) ☐ Definitely not

You have now finished all the questions. Thank you for contributing to this research project.
